# Supplementary material for: Clinical resistance to crenolanib in acute myeloid leukemia due to diverse molecular mechanisms
Source: Nat Commun. 2019 Jan 16;10:244. doi: 10.1038/s41467-018-08263-x (PMC6335421; doi:10.1038/s41467-018-08263-x)
Supplement: Supplementary file 1 — Supplementary Information [file 41467_2018_8263_MOESM1_ESM.docx]

**Supplementary Information**

**Clinical Resistance to Crenolanib in Acute Myeloid Leukemia Due to Diverse Molecular Mechanisms**

Zhang et al.

**Supplementary Table 1: Allelic burdens of non-D835 *FLT3* activation loop mutations during crenolanib treatment.**

| **ID** | **Patient** | **Prior TKI** | **Response** | **crenolanib treatment** | **D200N** | **K429E** | **Y572C** | **L601F** | **F691L** | **A833S** | **D839G** | **D839Y** | **N841K** | **Y842C** | **Y842D** | **delIns** | **Method** |
| --- | --- | --- | --- | --- | --- | --- | --- | --- | --- | --- | --- | --- | --- | --- | --- | --- | --- |
| **A11** | **1888** | **No** | **HI** | **Before** |  |  |  | **5.6%** |  |  |  |  |  |  |  |  | **Exome** |
|  |  |  |  | **After** |  |  |  | **9.5%** |  |  |  |  |  |  |  |  |  |
| **A04** | **1497** | **No** | **PR** | **Before** |  |  |  |  |  |  |  | **1.0%** |  |  |  |  | **Miseq** |
|  |  |  |  | **After** |  |  |  |  |  |  |  | **0.0%** |  |  |  |  |  |
| **A08** | **1550** | **No** | **CRi** | **Before** |  |  |  |  |  |  | **0.3%** | **0.3%** |  |  |  | **0.5%** | **Miseq** |
|  |  |  |  | **After** |  |  |  |  |  |  | **0.0%** | **0.0%** |  |  |  | **0.0%** |  |
| **MDA-120** | **1907** | **Yes** | **HI** | **Before** | **23.0%** |  |  |  |  |  |  |  |  |  |  |  | **Exome** |
|  |  |  |  | **After** | **13.8%** |  |  |  |  |  |  |  |  |  |  |  |  |
| **B11** | **1897** | **Yes** | **RD** | **Before** |  | **NA** |  |  |  |  |  |  |  |  |  |  | **Gene panel/** |
|  |  |  |  | **After** |  | **73.6%** |  |  |  |  |  |  |  |  |  |  | **Exome** |
| **B25** | **1904** | **Yes** | **RD** | **Before** |  |  |  |  | **17.1%** |  |  |  |  |  |  |  | **Exome** |
|  |  |  |  | **After** |  |  |  |  | **37.9%** |  |  |  |  |  |  |  |  |
| **A16** | **4088** | **Yes** | **RD** | **Before** |  |  | **28.8%** |  |  |  |  |  |  |  |  |  | **Miseq/** |
|  |  |  |  | **After** |  |  | **19.6%** |  |  |  |  |  |  |  |  |  | **Exome** |
| **B06** | **1500** | **Yes** | **RD** | **Before** |  |  |  |  | **0.0%** |  |  |  |  |  |  |  | **Miseq** |
|  |  |  |  | **After** |  |  |  |  | **37.6%** |  |  |  |  |  |  |  | **/Exome** |
| **B21** | **1908** | **Yes** | **CRi** | **Before** |  |  |  |  |  |  |  |  | **0.4%** |  |  |  | **Miseq** |
|  |  |  |  | **After** |  |  |  |  |  |  |  |  | **0.0%** |  |  |  |  |
| **A05** | **1498** | **Yes** | **RD** | **Before** |  |  |  |  |  | **3.3%** |  |  |  |  |  |  | **Miseq** |
|  |  |  |  | **After** |  |  |  |  |  | **0.0%** |  |  |  |  |  |  |  |
| **B19** | **1906** | **Yes** | **HI** | **Before** |  |  |  |  |  |  |  |  |  | **21.9%** |  |  | **Gene panel/** |
|  |  |  |  | **After** |  |  |  |  |  |  |  |  |  | **0.0%** |  |  | **Exome** |
| **B34** | **4190** | **Yes** | **RD** | **Before** |  |  |  |  |  |  |  |  |  |  | **9.4%** |  | **Exome** |
|  |  |  |  | **After** |  |  |  |  |  |  |  |  |  |  | **0.0%** |  |  |

DelIns: R834D835I836 (CGAGATATC) CGCCCC (RDI->RP).

**Supplementary Table 2:** List of queried hematopoietic genes.

| ABL1 | ARID1A | **ASXL1** | **ASXL2** | BCL10 | BCL11B | BCL6 | **BCOR** | **BCORL1** | **BIRC3** |
| --- | --- | --- | --- | --- | --- | --- | --- | --- | --- |
| BRAF | **BRCC3** | BTG1 | BTG2 | CARD11 | CBFB | CBL | CBLB | CCND2 | CCND3 |
| CD58 | CD70 | CD79A | CD79B | CDKN2A | CDKN2B | CEBPA | CHD2 | CNOT3 | CREBBP |
| CRLF2 | CSF1R | CSF3R | CTCF | **CUX1** | DDX3X | DIS3 | DNMT3A | EBF1 | EED |
| EP300 | ETNK1 | ETV6 | EZH2 | EZR | **FAM46C** | **FAS** | **FBXO11** | FBXW7 | FLT3 |
| **FOXP1** | FYN | GATA1 | GATA2 | GATA3 | GNA13 | GNAS | GNB1 | HIST1H1B | HIST1H1C |
| **HIST1H1D** | HIST1H1E | HIST1H3B | HLA-A | ID3 | IDH1 | IDH2 | IKBKB | **IKZF1** | **IKZF2** |
| **IKZF3** | IL7R | INTS12 | IRF4 | IRF8 | JAK1 | JAK2 | JAK3 | **JARID2** | **KDM6A** |
| KIT | KLHL6 | **KMT2A** | **KMT2C** | **KMT2D** | KRAS | **LEF1** | LRRK2 | **LTB** | **LUC7L2** |
| MALT1 | MAP2K1 | MAP3K14 | MED12 | MEF2B | MPL | MXRA5 | MYD88 | NF1 | NFE2 |
| **NOTCH1** | **NOTCH2** | NPM1 | NRAS | NTRK2 | NTRK3 | P2RY8 | **PAPD5** | **PAX5** | **PDS5B** |
| **PDSS2** | PHF6 | PIK3CA | **POT1** | POU2AF1 | POU2F2 | **PPM1D** | PRDM1 | PRPF40B | PRPF8 |
| PTEN | PTPN1 | PTPN11 | RAD21 | RAD21L1 | RBBP4 | RHOA | RIT1 | RPL10 | **RPL5** |
| RPS15 | RPS2 | RUNX1 | SETBP1 | SF3A1 | SF3B1 | **SGK1** | SH2B3 | SMC1A | SMC3 |
| **SOCS1** | SPRY4 | SRSF2 | **STAG1** | **STAG2** | STAT3 | STAT5A | STAT5B | STAT6 | **SUZ12** |
| **SWAP70** | **TBL1XR1** | TCF3 | **TET1** | TET2 | **TMEM30A** | TNF | TNFAIP3 | TNFRSF14 | TP53 |
| **TRAF3** | TYW1 | U2AF1 | U2AF2 | **UBR5** | WT1 | XBP1 | XPO1 | ZNF471 | ZRSR2 |

Gene with red bold font indicates that only frameshift, stop gain/loss and inframe insertion/deletion variants are considered for these genes.

**Supplementary Table 3: Summary of patient’s previous therapy.**

|  | TKI Naïve | Prior TKI | Combined Cohort |
| --- | --- | --- | --- |
|  | (n=19) | (n=31) | (n=50) |
|  | n (%) or [range] | n (%) or [range] | n (%) or [range] |
| Number of Prior Therapies |  |  |  |
| Median | 2 [1 - 7] | 3 [1 - 6] | 3 [1 - 7] |
| 1 | 9 (47) | 3 (10) | 12 (24) |
| 2 | 5 (26) | 5 (16) | 10 (20) |
| 3 | 2 (11) | 11 (35) | 13 (26) |
| 4 | 2 (11) | 7 (23) | 9 (18) |
| ≥ 5 | 1 (5) | 5 (16) | 6 (12) |
| Prior FLT3 TKI |  |  |  |
| Median | 0 [0] | 1 [1 - 4] | 1 [0 - 4] |
| Sorafenib | 0 (0) | 22 (71) | 22 (44) |
| Quizartinib | 0 (0) | 10 (32) | 10 (20) |
| Pexidartinib | 0 (0) | 3 (10) | 3 (6) |
| Gilteritinib | 0 (0) | 2 (6) | 2 (4) |
| Midostaurin | 0 (0) | 1 (3) | 1 (2) |

**Supplementary Figure 1**

**a**

**b**

**a**

**Figure legend:** **a,** Graph depicts mean ± SEM of colony numbers (upper panel) and or fold change of colony number (normalized to the mean colony number of no treatment, lower panel) for three replicates of mouse bone marrow cells transduced with retroviral vector expressing empty vector, *FLT3* WT or mutants treated with gradient concentrations of crenolanib for 10 days. Statistical analyses were performed using [one way ANOVA](http://homepages.inf.ed.ac.uk/rbf/CVonline/LOCAL_COPIES/SHUTLER2/node1.html) together with Dunn’s multiple comparisons tests comparing crenolanib treated cells to each individual untreated control and expressed as: * p<.05 and ** p<.01. **b,** Ba/F3 cells expressing *FLT3* compound mutations were grown in medium without IL-3. Cell numbers were determined as described in the materials and methods and shown in the graph.

**Supplementary Table 4. TKI naïve and pre-TKI patients demonstrate differential mutation profiles**

| **Gene** | **Percentage** | | | **Total number** | | |
| --- | --- | --- | --- | --- | --- | --- |
|  | **TCGA** | **TKI naïve** | **Pre-TKI** | **TCGA** | **TKI naïve** | **Pre-TKI** |
| FLT3 ITD | 69.60% | 47.4% | 38.7% | 56 | 19 | 31 |
| FLT3 TKD | 30.4 | 36.8% | 6.5% | 56 | 19 | 31 |
| FLT3ITD/TKD | 0% | 15.8% | 54.8% | 56 | 19 | 31 |
| *NPM1* | 51.8% | 42.1% | 51.6% | 56 | 19 | 31 |
| *DNMT3A* | 37.5% | 42.1% | 32.3% | 56 | 19 | 31 |
| *WT1* | 8.9% | 6.3% | 24.0% | 56 | 16 | 25 |
| *SF3B1* | 0.0% | 15.4% | 16.7% | 56 | 13 | 24 |
| *IDH1* | 7.1% | 0.0% | 16.1% | 56 | 19 | 31 |
| *RUNX1* | 5.4% | 6.3% | 20.0% | 56 | 16 | 25 |
| *NRAS* | 3.6% | 5.3% | 12.9% | 56 | 19 | 31 |
| *CSF3R* | 0.0% | 7.1% | 8.3% | 56 | 14 | 24 |
| *U2AF1* | 0.0% | 7.7% | 8.3% | 56 | 13 | 24 |
| *ASXL1* | 0.0% | 0.0% | 8.3% | 56 | 13 | 24 |
| *TET2_t* | 8.9% | 0.0% | 8.0% | 56 | 13 | 25 |
| *KRAS* | 0.0% | 0.0% | 6.5% | 56 | 19 | 31 |
| *IDH2* | 3.6% | 5.3% | 6.5% | 56 | 19 | 31 |
| *CBL* | 0.0% | 0.0% | 4.2% | 56 | 14 | 24 |
| *BCOR* | 1.8% | 0.0% | 4.2% | 56 | 13 | 24 |
| *STAG2* | 3.6% | 7.7% | 4.2% | 56 | 13 | 24 |
| *SRSF2* | 0.0% | 0.0% | 4.2% | 56 | 13 | 24 |
| *TET2_m* | NA | 18.8% | 4.0% | 56 | 16 | 25 |
| *JAK3* | 0.0% | 0.0% | 3.4% | 56 | 13 | 29 |
| *FLT3 F691* | 0% | 0.0% | 3.2% | 56 | 19 | 31 |
| *PTPN11* | 3.6% | 15.8% | 0.0% | 56 | 19 | 30 |
| *CEBPA* | 3.6% | 7.7% | 0.0% | 56 | 13 | 24 |
| *SETBP1* | 0.0% | 7.7% | 0.0% | 56 | 13 | 27 |
| *PPM1D* | 0.0% | 7.7% | 0.0% | 56 | 13 | 24 |
| *PHF6* | 1.8% | 7.7% | 0.0% | 56 | 13 | 24 |

*TET2-t*: *TET2* truncation mutation; *TET2_m*: *TET2* missense mutation

**Supplementary Figure 2**

**Figure legend**: **Patients with prior TKI treatment demonstrate differential gene mutation profiles as compared to TKI naïve patients.** Graph depicts log-transformed mean and 95% confidence intervals of odd ratios of gene mutation frequencies of pre-TKI group comparing to TKI naïve group. Statistical significance was analyzed using contingency table Fisher's exact test**.**

**Supplementary Table 5. Crenolanib good-responder and poor-responder demonstrate differential mutation profiles**

| **Gene** | **Percentage** | | | **Total number** | | |
| --- | --- | --- | --- | --- | --- | --- |
|  | **TCGA** | **Good-responder** | **Poor-responder** | **TCGA** | **Good-responder** | **Poor-responder** |
| *FLT3* ITD | 69.60% | 37.5% | 44.1% | 56 | 16 | 34 |
| *FLT3* TKD | 30.4 | 18.8% | 17.6% | 56 | 16 | 34 |
| *FLT3* ITD/TKD | 0% | 43.8% | 38.2% | 56 | 16 | 34 |
| *NPM1* | 51.8% | 56.3% | 44.1% | 56 | 16 | 34 |
| *DNMT3A* | 37.5% | 43.8% | 32.4% | 56 | 16 | 34 |
| *SF3B1* | 0.0% | 20.0% | 18.5% | 56 | 10 | 27 |
| *IDH1* | 7.1% | 0.0% | 14.7% | 56 | 16 | 34 |
| *NRAS* | 3.6% | 12.5% | 11.8% | 56 | 16 | 34 |
| *U2AF1* | 0.0% | 0.0% | 11.1% | 56 | 10 | 27 |
| *WT1* | 8.9% | 40.0% | 10.3% | 56 | 10 | 29 |
| *CSF3R* | 0.0% | 0.0% | 10.3% | 56 | 10 | 29 |
| *RUNX1* | 5.4% | 18.2% | 13.8% | 56 | 11 | 29 |
| *TET2_m* | NA | 0.0% | 9.3% | 56 | 11 | 29 |
| *STAG2* | 3.6% | 0.0% | 7.4% | 56 | 10 | 27 |
| *ASXL1* | 0.0% | 0% | 6.9% | 56 | 11 | 29 |
| *TET2_t* | 8.9% | 0.0% | 6.9% | 56 | 11 | 29 |
| *PTPN11* | 3.6% | 6.3% | 6.3% | 56 | 16 | 32 |
| *IDH2* | 3.6% | 0.0% | 5.9% | 56 | 16 | 34 |
| *KRAS* | 0.0% | 0.0% | 5.9% | 56 | 16 | 34 |
| *BCOR* | 1.8% | 0.0% | 3.7% | 56 | 10 | 27 |
| *SETBP1* | 0.0% | 0.0% | 3.7% | 56 | 10 | 27 |
| *PPM1D* | 0.0% | 0.0% | 3.7% | 56 | 10 | 27 |
| *SRSF2* | 0.0% | 0.0% | 3.7% | 56 | 10 | 27 |
| *PHF6* | 1.8% | 0.0% | 3.7% | 56 | 10 | 27 |
| *CBL* | 0.0% | 0.0% | 3.4% | 56 | 11 | 29 |
| *JAK3* | 0.0% | 0.0% | 3.1% | 56 | 10 | 32 |
| *FLT3* F691 | 0.0% | 0.0% | 2.9% | 56 | 16 | 34 |
| *CEBPA* | 3.6% | 0.0% | 0.0% | 56 | 10 | 27 |

non-responder: HI+RD; responder: CR/CRi+PR

**Supplementary Figure 3**

**Figure legend**: **Crenolanib good-responder and poor-responder groups demonstrate differential gene mutation profiles.** Graph depicts log-transformed mean and 95% confidence intervals of odd ratios of gene mutation frequencies of crenolanib non-responders comparing to crenolanib responders. Statistical significance was analyzed using contingency table Fisher's exact test**.**

**Supplementary Table 6. crenolanib response in patients with *TET2* mutations**

| **TET2 mutation** | **ID** | **Gene** | **Amino acid change** | **Clinical response** |
| --- | --- | --- | --- | --- |
| Frame shift | | | | |
|  | B24 | *TET2* | Q1825* | RD |
|  |  |  | Q860X |  |
|  | B18 | *TET2* | GLKEGR1361G | RD |
| missense | |  |  |  |
|  | A08 | *TET2* | D1143V | CRI |
|  | B21 | *TET2* | R1214W | PR |
|  |  |  | V1718L |  |
|  | A12 | *TET2* | H1380Y | RD |
|  | B19 | *TET2* | Y867H | PR |
|  |  |  | P1723S |  |
| Combined |  |  |  |  |
|  | A05 | *TET2* | R1214W | RD |
|  |  | *TET2* | S907X |  |
|  | B11 | *TET2* | R1261L | RD |
|  |  | *TET2* | S1556X |  |
|  | B13 | *TET2* | P419X | HI |

**
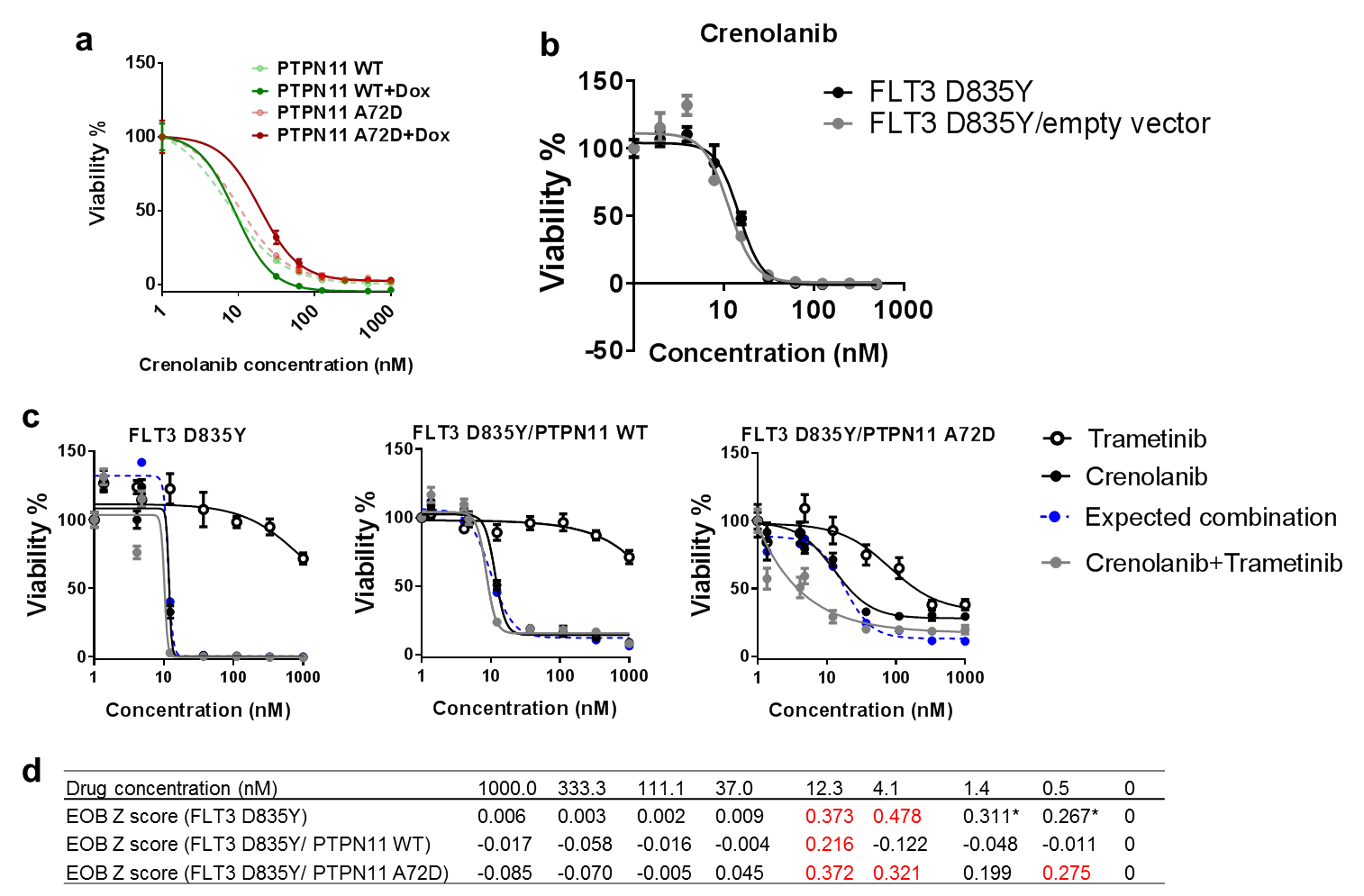
Supplementary Figure 4**

**Figure legend: Crenolanib and trametinib combination therapy demonstrate synergistic toxicity****. a,** Representative graph depicts mean ± SEM of cell viability of MV4-11 cells expressing a doxycycline inducible PTPN11 WT or PTPN11 A72D vector treated with dose gradients of crenolanib in the presence or absence of doxycycline (Dox, 1ug/ml) for 72h determined by MTS assay as described in Materials and Methods. **b,** Graph depicts mean ± SEM of cell viability of Ba/F3 cells expressing FLT3 D835Y or FLT3 D835Y in combination with an empty vector treated with dose gradients of crenolanib for 72h determined by MTS assay as described in Materials and Methods. **c,** Graph depicts mean ± SEM of cell viabilities of Ba/F3 cells expressing FLT3 D835Y along or with PTPN11 WT or PTPN11 72D compound mutation treated with crenolanib, trametinib, or two drug equal concentration combination. The predicted viability of crenolanib and trametinib combination is shown in blue according to EOB calculation as defined in the materials and methods. **d,** The table summarizes the EOB z score of crenolanib/trametinib combination at each concentration. * indicates that the drug combination effect is smaller than the HSA effect. Red highlights that crenolanib+trametinib combination demonstrates strongly synergistic effect.

**Supplementary Figure 5**

**Figure legend**: *Flt3*^ITD^, *Flt3*^ITD^; *Tet2*^+/-^ and healthy mouse lineage negative stem cells were cultured in methylcellulose medium in presence of gradient concentrations of crenolanib or azacytidine as described in the Materials and Methods for 10 days. Cells were harvested and washed and stained with Sca-1 antibody. Graph depicts mean ± SEM of percentages of Sca-1 positive cells from three replicates.

**
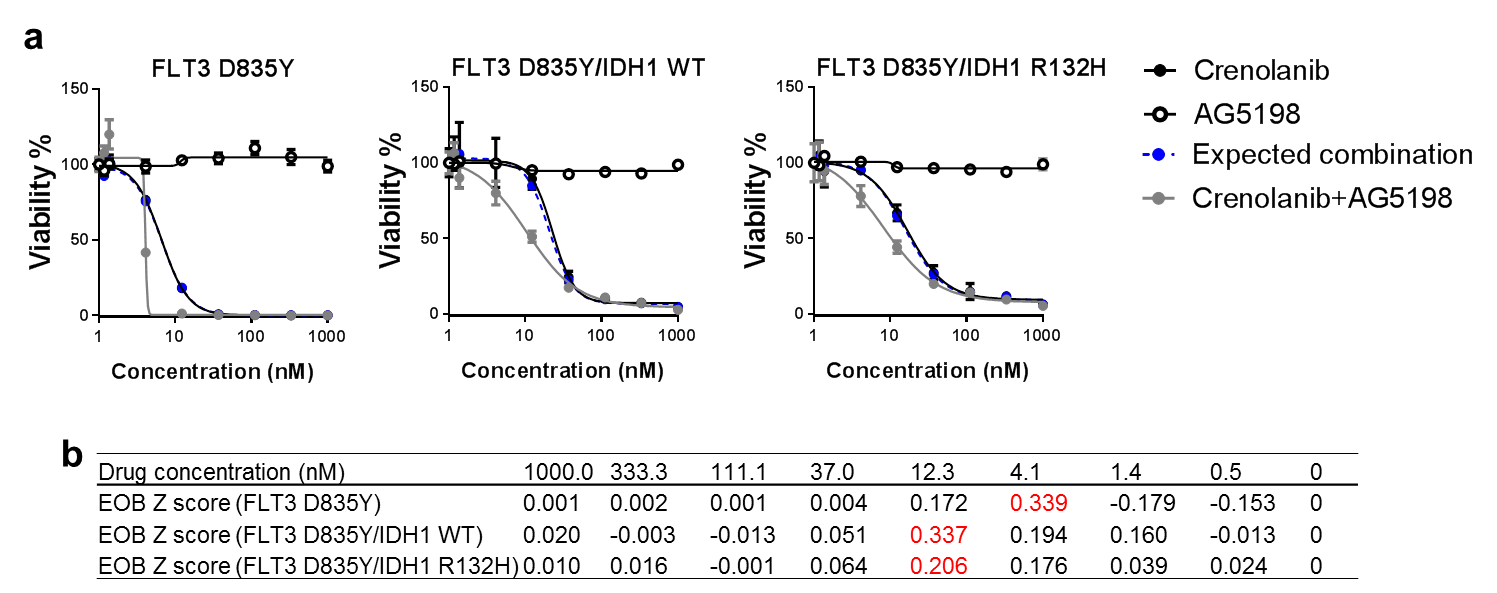
Supplementary Figure 6**

**Figure legend: Crenolanib and trametinib combination therapy demonstrate synergistic toxicity. a,** Graph depicts mean ± SEM of cell viabilities of Ba/F3 cells expressing *FLT3* D835Y along or with *PTPN11* WT or *PTPN11* 72D compound mutation treated with crenolanib, trametinib, or two drug equal concentration combination. The predicted viability of crenolanib and trametinib combination is shown in blue according to EOB calculation as defined in the materials and methods. **b,** The table summarizes the EOB z score of crenolanib/trametinib combination at each concentration. * indicates that the drug combination effect is smaller than the HSA effect. Red highlights that crenolanib+trametinib combination demonstrates strongly synergistic effect.

**Supplementary Figure 7**


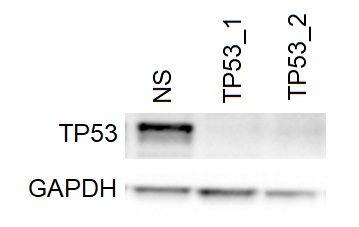


36

50

**Figure legend: TP53 knockout validation.** Representative immunoblot image confirms knockout of TP53 using CRISPR /Cas9 system as described in the methods. TP53_1: sgRNA1 targeting *TP53*; TP53_2: sgRNA2 targeting *TP53*; NS: a non-specific targeting sgRNA control.
