# Supplementary material for: Clinical resistance to crenolanib in acute myeloid leukemia due to diverse molecular mechanisms
Source: Nat Commun. 2019 Jan 16;10:244. doi: 10.1038/s41467-018-08263-x (PMC6335421; doi:10.1038/s41467-018-08263-x)
Supplement: Supplementary file 3 — Description of Additional Supplementary Files [file 41467_2018_8263_MOESM3_ESM.docx]

Description of Additional Supplementary Files

Supplementary Data 1. Miseq data

Supplementary Data 2. Exome and validation sequencing

Supplementary Data 3. Summary of all patients' information

Supplementary Data 4. Summary of patient's previous therapies
