# Supplementary material for: Clinical resistance to crenolanib in acute myeloid leukemia due to diverse molecular mechanisms
Source: Nat Commun. 2019 Jan 16;10:244. doi: 10.1038/s41467-018-08263-x (PMC6335421; doi:10.1038/s41467-018-08263-x)
Supplement: Supplementary file 8 — Reporting Summary [file 41467_2018_8263_MOESM8_ESM.pdf]

nature research

Corresponding author(s): Jeffrey Tyner

Reporting Summary

Nature Research wishes to improve the reproducibility of the work that we publish. This form provides structure for consistency and transparency in reporting. For further information on Nature Research policies, see Authors & Referees and the Editorial Policy Checklist.

Statistical parameters

When statistical analyses are reported, confirm that the following items are present in the relevant location (e.g. figure legend, table legend, main text, or Methods section).

n/a	Confirmed	
☐	☒	The exact sample size (*n*) for each experimental group/condition, given as a discrete number and unit of measurement
☐	☒	An indication of whether measurements were taken from distinct samples or whether the same sample was measured repeatedly
☐	☒	The statistical test(s) used AND whether they are one- or two-sided<br>*Only common tests should be described solely by name; describe more complex techniques in the Methods section.*
☐	☒	A description of all covariates tested
☐	☒	A description of any assumptions or corrections, such as tests of normality and adjustment for multiple comparisons
☐	☒	A full description of the statistics including central tendency (e.g. means) or other basic estimates (e.g. regression coefficient) AND variation (e.g. standard deviation) or associated estimates of uncertainty (e.g. confidence intervals)
☐	☒	For null hypothesis testing, the test statistic (e.g. *F*, *t*, *r*) with confidence intervals, effect sizes, degrees of freedom and *P* value noted<br>*Give P values as exact values whenever suitable.*
☒	☐	For Bayesian analysis, information on the choice of priors and Markov chain Monte Carlo settings
☒	☐	For hierarchical and complex designs, identification of the appropriate level for tests and full reporting of outcomes
☒	☐	Estimates of effect sizes (e.g. Cohen's *d*, Pearson's *r*), indicating how they were calculated
☐	☒	Clearly defined error bars<br>*State explicitly what error bars represent (e.g. SD, SE, CI)*

Our web collection on statistics for biologists may be useful.

Software and code

Policy information about availability of computer code

Data collection	Data was collected using Illumina Real Time Analysis (RTA) 1.18; Data was assembled to fastq files using Illumina Bcl2Fastq2 v2.xx
Data analysis	For whole exome sequencing, we used the Illumina Nextera capture probes and protocol (12 samples per capture group with each sample run on 3,5 or 6 lanes) with libraries run on a HiSeq 2500 using paired end 100 cycle protocols. Initial data processing and alignments were performed using our in-house workflows that we describe here briefly. For each flowcell and each sample, the FASTQ files were aggregated into single files for read 1 and 2. BWA MEM version 0.7.10-r78957 was used to align the read pairs for each sample-lane FASTQ file. As part of this process, the flowcell and lane information was kept as part of the read group of the resulting SAM file. The Genome Analysis Toolkit (v3.3) and the bundled Picard (v1.120.1579) were used58 for alignment post-processing. The files contained within the Broad's bundle 2.8 were used including their version of the build 37 human genome. The following steps were performed per sample-lane SAM file: (1) Sorting and conversion to BAM via SortSam; (2) MarkDuplicates was run, marking both lane level standard and optical duplicates; (3) Read realignment around indels from the reads RealignerTargetCreator/IndelRealigner; (4) Base Quality Score Recalibration. The resulting BAM files were then aggregated by sample and an additional round of MarkDuplicates and indel realignment was carried out at the sample level. For genotyping, SNV and small indels were called using the UnifiedGenotyper2 and VarScan23. Additionally, SNVs were called by MuTect4. Each VCF file was annotated using the Variant Effect Predictor v835 against GRCh37.<br>For the validation sequencing, libraries were created and hybridized using custom designed Nimblegen (SeqCap EZ) probes covering genes of interest containing both known variants as well as novel recurrent variants seen in other AML samples sequenced in-house. Because of the smaller library size, we ran 12 samples per lane. Paired-end 100 base reads were generated, aligned and post-processed using the WES protocol described above. For each unique variant position observed in the WES data, the number of reads supporting

each observed allele in the validation library was determined using bam-readcount6. A variant was considered to be 'validated' by sequencing if there were at least 3 reads supporting the called variant.

For manuscripts utilizing custom algorithms or software that are central to the research but not yet described in published literature, software must be made available to editors/reviewers upon request. We strongly encourage code deposition in a community repository (e.g. GitHub). See the Nature Research guidelines for submitting code & software for further information.

Data

Policy information about availability of data

All manuscripts must include a data availability statement. This statement should provide the following information, where applicable:

- Accession codes, unique identifiers, or web links for publicly available datasets
- A list of figures that have associated raw data
- A description of any restrictions on data availability

All raw and processed sequencing data, along with relevant clinical annotations are submitted to dbGaP and Genomic Data Commons. The raw data for clinical annotations, and variant calls that underlie all figures in this manuscript are found in the Supplementary Information. In addition, all data can be accessed and queried through our online, interactive user interface, Vizome, at www.vizome.org.

Field-specific reporting

Please select the best fit for your research. If you are not sure, read the appropriate sections before making your selection.

☒ Life sciences ☐ Behavioural & social sciences ☐ Ecological, evolutionary & environmental sciences

For a reference copy of the document with all sections, see nature.com/authors/policies/ReportingSummary-flat.pdf

Life sciences study design

All studies must disclose on these points even when the disclosure is negative.

Sample size

Samples were obtained according to the Declaration of Helsinki under IRB-approved protocols from patients who were enrolled on Phase II clinical trials of crenolanib (NCT 01522469 and NCT 01657682) in relapsed or refractory AML at University of Texas Southwestern Medical Center and MD Anderson Cancer Center. The total number of patient is 69. The sample size were determined by the affiliated clinical trial study described elsewhere (2016 ASH Abstract #7008.).

Data exclusions

Since no paired normal tissue controls were available, we compiled a list genes associated with human hematologic cancers according to these two papers (Jaiswal et al., NEJM 2014; and AML TCGA, NEJM 2013). In total 170 genes were selected (Supplementary Table 2). We used the global filtering as previous described (Tyner et al., Nature 2018). On top of that, the following filters were used: 1) excluding variants found in more than 0.1% of ExAC samples; and excluding variants found in normal samples from more than 20% BeatAML normal controls; 2) including variant types: Missense; Frameshift; Stop gain/loss; Inframe insertion/deletion; Protein altering; and Tandem duplication for 127 genes list in Supplementary table 1 (regular black font). 4) In addition, only frameshift, stop gain/loss and Inframe insertion/deletion variants are considered for the following 43 genes (bold red font).
The 127 genes include:
ABL1
ARID1A
BCL10
BCL11B
BCL6
BRAF
BTG1
BTG2
CARD11
CBFB
CBL
CBLB
CCND2
CCND3
CD58
CD70
CD79A
CD79B
CDKN2A
CDKN2B
CEBPA
CHD2
CNOT3
CREBBP
CRLF2
CSF1R
CSF3R

CTCF
DDX3X
DIS3
DNMT3A
EBF1
EED
EP300
ETNK1
ETV6
EZH2
EZR
FBXW7
FLT3 (To search in www.vizome.org, please use FLT3 and FLT3_ITD to get the list of all variants)
FYN
GATA1
GATA2
GATA3
GNA13
GNAS
GNB1
HIST1H1B
HIST1H1C
HIST1H1E
HIST1H3B
HLA-A
ID3
IDH1
IDH2
IKBKB
IL7R
INTS12
IRF4
IRF8
JAK1
JAK2
JAK3
KIT
KLHL6
KRAS
LRRK2
MALT1
MAP2K1
MAP3K14
MED12
MEF2B
MPL
MXRA5
MYD88
NF1
NFE2
NPM1
NRAS
NTRK2
NTRK3
P2RY8
PHF6
PIK3CA
POU2AF1
POU2F2
PRDM1
PRPF40B
PRPF8
PTEN
PTPN1
PTPN11
RAD21
RAD21L1
RBBP4
RHOA
RIT1
RPL10
RPS15
RPS2
RUNX1
SETBP1

SF3A1
SF3B1
SH2B3
SMC1A
SMC3
SPRY4
SRSF2
STAT3
STAT5A
STAT5B
STAT6
TCF3
TET2
TNF
TNFAIP3
TNFRSF14
TP53
TYW1
U2AF1
U2AF2
WT1
XBP1
XPO1
ZNF471
ZRSR2
FLT3-ITD
The 43 genes include:
ASXL1
ASXL2
BCOR
BCORL1
BIRC3
BRCC3
CUX1
FAM46C
FAS
FBXO11
FOXP1
HIST1H1D
IKZF1
IKZF2
IKZF3
JARID2
KDM6A
KMT2A
KMT2C
KMT2D
LEF1
LTB
LUC7L2
NOTCH1
NOTCH2
PAPD5
PAX5
PDS5B
PDSS2
POT1
PPM1D
RPL5
SGK1
SOCS1
STAG2
SUZ12
SWAP70
TBL1XR1
TET1
TMEM30A
TRAF3
UBR5
STAG1

Replication

All data analysis pipelines filtering, exclusions and quality control steps are described above and in detail in the Methods. Each analytical approach and result was replicated successfully.

Randomization	All samples were assigned numerical identifications with no association to any features of the sample, and for all sequencing batches samples were randomized into capture library groups and flow cells.
Blinding	All samples were assigned numerical identifications that bore no relevance to sample features or attributes, and all data analyses were performed using these de-identified specimen ID numbers

Reporting for specific materials, systems and methods

Materials & experimental systems

n/a	Involved in the study	
☒	☐	Unique biological materials
☐	☒	Antibodies
☐	☒	Eukaryotic cell lines
☒	☐	Palaeontology
☐	☒	Animals and other organisms
☐	☒	Human research participants

Methods

n/a	Involved in the study	
☒	☐	ChIP-seq
☒	☐	Flow cytometry
☒	☐	MRI-based neuroimaging

Antibodies

Antibodies used	TP53 antibody from Cell Signaling (#2524), GAPDH antibody from Thermo Fisher (#AM4300) were used.
Validation	For TP53 antibody, a protein band with a molecular weight about 53KD, which matched the expected p53 protein were detected. For GAPDH antibody, a protein band with a molecular weight about 39-40KD, which matched the expected GAPDH protein were detected.

Eukaryotic cell lines

Policy information about cell lines

Cell line source(s)	HEK 293T/17 cells were provided by Dr. Richard Van Etten. Ba/F3 cells were from ATCC. Molm13, Molm14 and MV4-11 cells were from DSMZ.
Authentication	The cell lines were authenticated by internal FLT3-ITD PCR and small inhibitor screening as well as short tandem repeat analysis.
Mycoplasma contamination	Mycoplasma contamination was routinely tested (Once per month). Only mycoplasma free cells were used in the experiments.
Commonly misidentified lines (See ICLAC register)	No misidentified lines according to the list were used

Animals and other organisms

Policy information about studies involving animals; ARRIVE guidelines recommended for reporting animal research

Laboratory animals	WT, Flt3ITD and Flt3ITD;Tet2+/– transgenic BALB/c mice were used.
Wild animals	BALB/c mice from The Jackson Lab (#001026)
Field-collected samples	Female for WT BALB/c mice<br>mix gender for transgenic mice<br>Genotype

Human research participants

Policy information about studies involving human research participants

Population characteristics	This is all documented in great detail in the Methods and in Supplementary Information. Below is a list of covariate population characteristics:<br>Patient ID<br>Age<br>Gender

Prior Regimen
Pre-TKI status
FLT3_Mutation
Reponse
Karyotype at baseline
Exome sequencing SampleID
Timepoint
Sample type
Antecedent Hematologic Disorder (AHD)
PB Blast%
BM Blast%
FLT3 D835 VAF(%)
Non-D835 FLT3 tyrosine kinase domain mutation VAF (%)
FLT3-ITD VAF (%)
Mutation
Mutation VAF (%)
NPM1
DNMT3A
FLT3 F691L
FLT3 K429E
TET2
NRAS
KRAS
PTPN11
CBL
NF1
ABL1
TP53
PPMD1
IDH1
IDH2
STAG2
RAD21L1
ASXL1
ASXL2
CEBPa
WT1
RUNX1
CBFB
SF3B1
CCND3
U2AF1
SRSF2
BCOR
BCORL1
PHF6
SETBP1
CTCF
CSF3R
JAK3

Recruitment

Patients were relapsed or refractory AML patients that were enrolled in phase II clinical trials of crenolanib (NCT 01522469 or NCT 01657682) at University of Texas Southwestern Medical Center or MD Anderson Cancer Center.
